# Supplementary material for: Genome-Wide Identification, Expression, and Molecular Characterization of the CONSTANS-like Gene Family in Seven Orchid Species
Source: Int J Mol Sci. 2023 Nov 27;24(23):16825. doi: 10.3390/ijms242316825 (PMC10706594; doi:10.3390/ijms242316825)
Supplement: Supplementary file 1 [file ijms-24-16825-s001.zip › Supplementary Figure_S1.pdf]

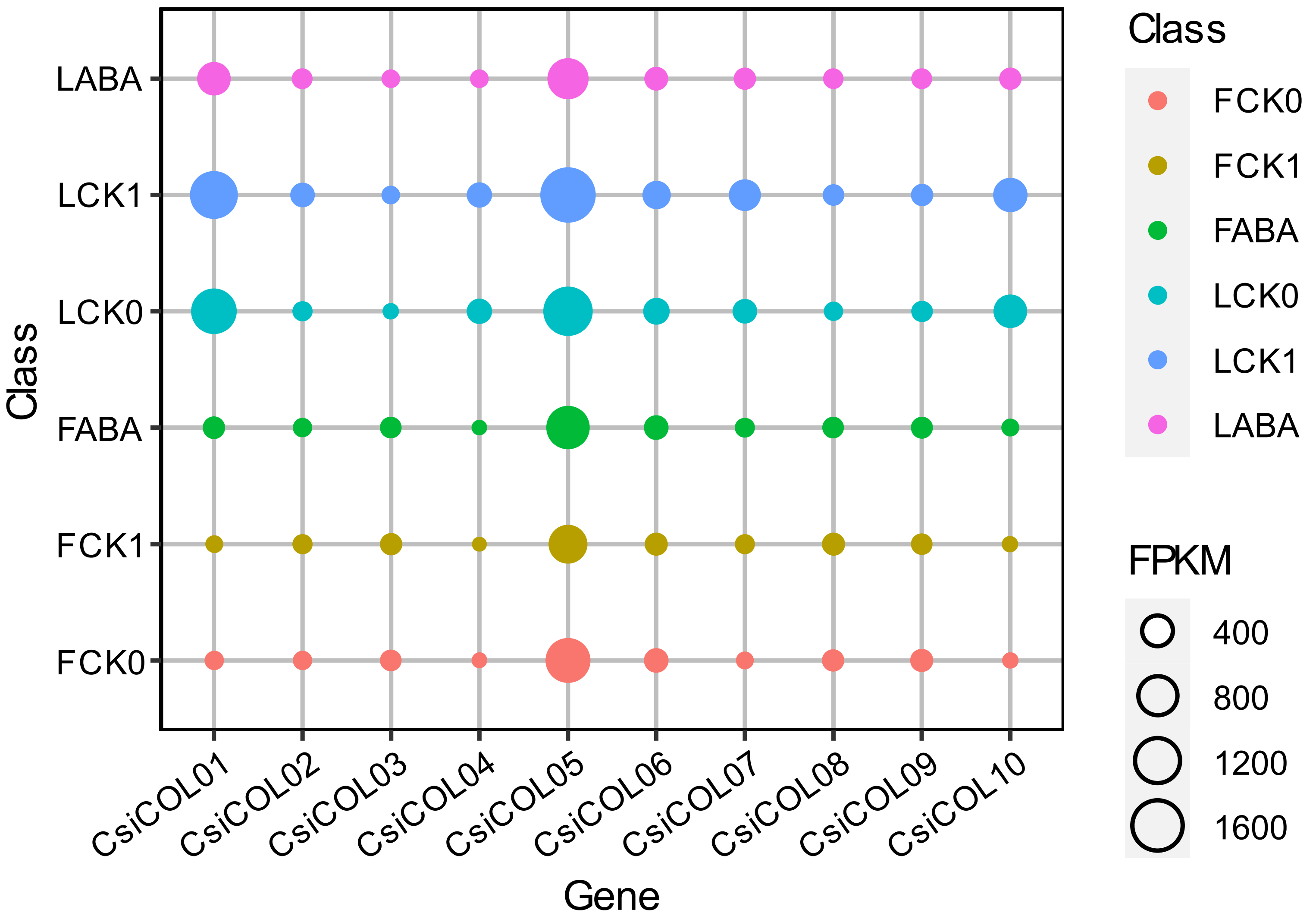

Supplementary Figure S1. Expression levels of *CsiCOLs* in flower and leaf of *C. sinense* before and after ABA treatment. LCK0, Initial leaf control samples; LCK1, Control sample of leaves after one month; LABA, Leaf samples after one month of ABA treatment. FCK0, Initial flower control samples; FCK1, Control sample of leaves after one month; FABA, flower samples after one month of ABA treatment.
